# Impaired Glucose Metabolism Is Associated with Visit-to-Visit Blood Pressure Variability in Participants without Cardiovascular Disease

**DOI:** 10.1155/2018/5126270

**Published:** 2018-04-23

**Authors:** Nobuo Sasaki, Ryoji Ozono, Saeko Fujiwara, Ryo Maeda, Yasuki Kihara

**Affiliations:** ^1^Health Management and Promotion Center, Hiroshima Atomic Bomb Casualty Council, Hiroshima, Japan; ^2^Department of General Medicine, Hiroshima University Graduate School of Biomedical and Health Sciences, Hiroshima, Japan; ^3^Department of Cardiovascular Medicine, Hiroshima University Graduate School of Biomedical and Health Sciences, Hiroshima, Japan

## Abstract

We evaluated data from 10,088 participants without cardiovascular disease (CVD) who underwent 75 g oral glucose tolerance tests and had more than four visits during the first 5 years following the test to investigate the association between impaired glucose metabolism and visit-to-visit blood pressure (BP) variability. Participants were classified into groups of normal glucose tolerance (NGT), impaired fasting glucose (IFG), impaired glucose tolerance (IGT), and diabetes. Visit-to-visit BP variability was estimated for each individual using standard deviation (SD) and coefficients of variation (CV, defined as SD/mean). SDs and CVs of systolic BP (SBP) values were divided into quartiles. The samples falling in the highest quartile were considered as having high SD/CV. The adjusted odds ratio (OR) for high SD of SBP in the IFG (OR, 1.39; *P* < 0.003), IGT (OR, 1.26; *P* < 0.001), and diabetes (OR, 1.54; *P* < 0.001) groups was significantly higher than that for high SD of SBP in the NGT group. Similarly, the OR for high CV of SBP in the IGT and diabetes groups was significantly higher than that for high CV of SBP in the NGT group. In participants without CVD, impaired glucose metabolism may modulate visit-to-visit BP variability.

## 1. Introduction

Visit-to-visit blood pressure (BP) variability reflects arterial stiffness and may be predictive of cardiovascular morbidity and mortality [1, 2]. Several studies in patients with diabetes have demonstrated a significant association between visit-to-visit BP variability and incident cardiovascular disease (CVD) [3–5]. Glucose fluctuation could contribute to oxidative stress, renin–angiotensin system activation, inflammation initiation, and endothelial dysfunction [6, 7], resulting in the development of atherosclerosis and increased arterial stiffness. In addition, neuropathy in diabetes could contribute to baroreflex sensitivity [8], which may modulate visit-to-visit BP variability [9]. These data suggest that visit-to-visit BP variability is a predictor of cardiovascular outcomes and may also be associated with the pathophysiology of diabetes. However, whether glucose metabolism modulates visit-to-visit BP variability remains unclear. Little is known about this BP variability particularly in participants with intermediate hyperglycemia (i.e., those with impaired fasting glucose [IFG] or impaired glucose tolerance [IGT]) [10, 11].

In the present study, we investigated the association between visit-to-visit BP variability and the status of glucose metabolism, alternating from normal glucose tolerance (NGT) to intermediate hyperglycemia including IFG, IGT, and diabetes in a large-scale population without CVD.

## 2. Methods

### 2.1. Study Participants

The present study used data collected from the Hiroshima Aging, Blood pressure, and Diabetes study (Hiroshima ABD study), a cross-sectional and longitudinal study that examined the interrelationship among aging, blood pressure, glucose metabolism, and cardiovascular outcomes. Potential participants were recruited between April 1988 and March 2012, after undergoing annual health examinations at the Health Management and Promotion Center of Hiroshima Atomic Bomb Casualty Council. Potential participants were considered eligible if they (1) were aged ≥18 years, (2) had no symptomatic heart failure, (3) had no history of treatment for diabetes, and (4) agreed to undergo the 75 g oral glucose tolerance test (OGTT). We asked all participants about their regular medications and medical histories, including treatment for hypertension, diabetes, dyslipidemia, and CVD, and information about their drinking and smoking habits. The present study reports on 10,088 participants (4,938 men and 5,150 women with mean age ± standard deviation [SD] of 65.7 ± 7.3 years and mean body mass index [BMI] ± SD of 23.2 ± 3.1 kg/m2) enrolled between April 1988 and March 2007. Included participants had no current or a prior history of CVD and had more than four visits during the first 5 years for health examinations. Among the 10,088 participants, 4,759 (47%) had hypertension (defined as taking antihypertensive medications [*n* = 2,103] and/or having systolic BP [SBP] ≥ 140 mmHg and/or diastolic BP [DBP] ≥ 90 mmHg); 1,072 (11%) had dyslipidemia (defined as taking antihyperlipidemic medications); 1,731 (17%) were current smokers (defined as having a current smoking habit regardless of the number of cigarettes smoked per day); and 4,388 (43%) were habitual drinkers (defined as drinking alcohol for ≥5 days per week, regardless of the amount consumed) [12]. Informed consent was obtained from all participants during their health examinations. Ethical approval for this study was obtained from the Hiroshima Atomic Bomb Casualty Council committee on the ethics of human research.

### 2.2. Measurements

The 75 g OGTT was conducted in the morning after an overnight fast. Blood samples were drawn just before and 30, 60, and 120 min after loading of glucose. Plasma glucose was measured using the enzymatic method. BP was measured using a standard sphygmomanometer in a seated position on a chair with back support and arm support at heart level after resting for >5 min prior to undergoing OGTT. Follow-up BP was measured under the same conditions during the annual health examinations. Mean BP was defined as the average of BP values measured on the baseline and follow-up visits. Visit-to-visit BP variability for each individual was defined by between-visit SD and coefficient of variation (CV). CV was defined as SD/mean. SDs and CVs of SBP and DBP values were divided into quartiles. The samples falling in the highest quartile were considered as having high SD/CVs and those in the remaining three quartiles as having low SD/CVs.

### 2.3. Participant Classification

We divided the participants into four groups according to their glycemic status as defined by the 2006 World Health Organization criteria [10] and the Japan Diabetes Society criteria [11]: (1) NGT, defined as fasting plasma glucose (FPG) < 6.1 mmol/l (110 mg/dl) and 2 h postload glucose < 7.8 mmol/l (140 mg/dl); (2) IFG, defined as FPG ≥ 6.1 and < 7.0 mmol/l (126 mg/dl) and 2 h postload glucose < 7.8 mmol/l; (3) IGT, defined as FPG < 7.0 mmol/l and 2 h postload glucose ≥ 7.8 and < 11.1 mmol/l; and (4) diabetes, defined as FPG ≥ 7.0 and/or 2 h postload glucose ≥ 11.1 mmol/l.

### 2.4. Statistical Analysis

Continuous variables were expressed as mean ± SD. Differences among the four groups (i.e., NGT, IFG, IGT, and diabetes) were evaluated using analysis of variance. Fisher's least-significant-difference method was used for multiple comparisons. Categorical variables were summarized as percentages and were analyzed using the chi-square test. The odds ratios (ORs) of high SD/CV of SBP and DBP were calculated using univariate and multivariate logistic regression analysis, adjusting for age, gender, BMI, habitual drinker (yes or no), current smoker (yes or no), taking antihypertensive medication (yes or no), dyslipidemia (yes or no), and number of visits. A *P* value < 0.05 was considered statistically significant. All statistical analyses were performed using the JMP 10 statistical software (SAS Institute, Inc., Cary, NC, USA).

## 3. Results

Clinical characteristics of the study population are presented in Table 1. The mean age was slightly lower in the IFG and diabetes groups and higher in the IGT group than in the NGT group. BMI was highest in the diabetes group, followed in descending order by IGT, IFG, and NGT groups. Table 1 shows the study population characteristics when participants are divided into the high and low SBP SD groups.

Table 2 shows group differences in BP measurements and BP variability. We found that SD of both SBP and DBP was significantly higher in the IFG, IGT, and diabetes groups than in the NGT group. Similarly, CV of SBP was significantly higher in both IGT and diabetes groups than in the NGT group. There was no significant difference in CV of DBP among the four groups.

Table 3 shows the results from univariate and multivariate logistic regression analyses. After adjusting for age, sex, BMI, smoking, drinking, taking antihypertensive medication, dyslipidemia, and number of visits, the OR for high SD of SBP in the IFG (OR, 1.39; 95% confidence interval [CI], 1.12–1.73; *P* < 0.005), IGT (OR, 1.26; 95% CI, 1.13–1.41; *P* < 0.001), and diabetes (OR, 1.54; 95% CI, 1.35–1.76; *P* < 0.001) groups was significantly higher than that for high SD of SBP in the NGT group. The OR for high CV of SBP in the IGT (OR, 1.15; 95% CI, 1.03–1.28; *P* < 0.05) and diabetes (OR, 1.35; 95% CI, 1.19–1.54; *P* < 0.001) groups (but not the IFG group) was significantly higher than that for high CV of SBP in the NGT group.

## 4. Discussion

In this study, we demonstrated higher visit-to-visit SBP variability in participants with intermediate hyperglycemia (including IFG and IGT) compared with those with NGT. The rate of high visit-to-visit SBP variability during the first 5 years gradually increased, starting with low increases in NGT cases, moderate increases in intermediate hyperglycemia cases, and highest increases in diabetes cases after the baseline 75 g OGTT. These results suggest that, in participants without CVD, impaired glucose metabolism may modulate visit-to-visit BP variability. In particular, we found that IGT, as well as diabetes, was significantly associated with high SD and CV of SBP. Although IGT has been reported to be a significant risk factor for CVD [13], the underlying mechanisms are not fully understood. Our results indicate that visit-to-visit BP variability may be involved in adverse consequences of IGT.

Although the determinants of visit-to-visit BP variability are not completely understood, arterial stiffness is thought to be one of the key factors [14]. It is well known that arterial stiffness is prevalent in patients with diabetes [15]. Furthermore, several studies have reported that intermediate hyperglycemia, including IFG and IGT, is associated with arterial stiffness [16, 17]. Pietri et al. reported that pulse wave velocity, a marker of arterial stiffness, gradually increased with increasing degree of abnormal glucose metabolism ranging from normal to intermediate hyperglycemia and diabetes [18]. Therefore, our results may be partly explained by increased arterial stiffness that is common in patients with intermediate hyperglycemia and diabetes. Disturbances of baroreflex function also exaggerate visit-to-visit BP variability [9]. Ruiz et al. reported lower baroreflex sensitivity in patients with diabetes compared with nondiabetic participants, and the disturbance in baroreflex sensitivity does not appear to depend on carotid artery atherosclerosis [8]. In another study, Wu et al. reported that participants with IGT had impaired baroreflex sensitivity compared to NGT [19]. Thus, the disturbances in baroreflex function may partly explain the observed higher visit-to-visit BP variability in participants with IGT in this study.

In patients with diabetes, most studies reported visit-to-visit BP variability as an independent risk factor for macro- and microvascular complications [3, 5, 20, 21]. In contrast, a recent large-scale observational study revealed that higher visit-to-visit BP variability was associated with incident diabetes [22]. Although the underlying mechanisms remain unclear, these data have led to the hypothesis that visit-to-visit BP variability has an adverse effect on glucose metabolism and is associated with contributing factors for the onset of diabetes. Conversely, our study provides evidence suggesting that impaired glucose metabolism may modulate visit-to-visit BP variability. Further studies are needed to clarify the interaction between visit-to-visit BP variability and impaired glucose metabolism.

This study has some limitations. First, the types of antihypertensive agents used were not recorded for participants currently under treatment for hypertension and the effects on visit-to-visit BP variability may differ based on the use of different antihypertensive agents. Second, participants with CVD were excluded based on careful interview inquiring about regular medications, medical history, and a physical examination. Nonetheless, we cannot rule out the possibility that the present study population included participants with subclinical CVD, given that we did not assess cardiac function using more objective methods.

In conclusion, our results suggested that impaired glucose metabolism may modulate visit-to-visit BP variability. Intermediate hyperglycemia, especially IGT, was associated with visit-to-visit BP variability. This may explain the adverse cardiovascular consequences observed in patients with IGT.

## Figures and Tables

**Table 1:** Characteristics of participants with normal glucose tolerance, impaired glucose metabolism, and diabetes.

		NGT	IFG	IGT	DM	*P*
*N*		5235	472	2795	1586	
Mean age	(years)	65.7 ± 7.4	64.8 ± 7.6^c^	66.3 ± 7.2^a^	65.1 ± 7.5^b^	<0.001
Female	[*n* (%)]	2887 (55)	197 (42)	1340 (48)	726 (46)	<0.001
BMI	(kg/m2)	22.6 ± 2.9	23.4 ± 3.1^a^	23.7 ± 3.2^a^	24.2 ± 3.3^a^	<0.001
Current smoker	[*n* (%)]	838 (16)	80 (17)	486 (17)	327 (21)	<0.001
Habitual drinker	[*n* (%)]	2174 (42)	250 (53)	1263 (45)	701 (44)	<0.001
Hypertension	[*n* (%)]	2046 (39)	261 (55)	1497 (54)	955 (60)	<0.001
Antihypertensive medication	[*n* (%)]	891 (17)	110 (23)	709 (25)	393 (25)	<0.001
Dyslipidemia	[*n* (%)]	539 (10)	41 (9)	343 (12)	149 (9)	0.005

Classified by SBP SD						

High SD						
*N*		1150	133	754	485	
Mean age	(years)	66.7 ± 7.5	66.5 ± 7.5	67.1 ± 7.2	65.6 ± 7.1^c^	0.005
Female	[*n* (%)]	624 (54)	59 (44)	396 (53)	213 (44)	<0.001
BMI	(kg/m2)	22.5 ± 2.9	23.6 ± 3.4^a^	23.7 ± 3.3^a^	24.0 ± 3.3^a^	<0.001
Current smoker	[*n* (%)]	212 (18)	22 (17)	134 (18)	118 (24)	0.020
Habitual drinker	[*n* (%)]	490 (43)	76 (57)	314 (42)	208 (43)	0.010
Hypertension	[*n* (%)]	645 (56)	97 (73)	515 (68)	351 (72)	<0.001
Antihypertensive medication	[*n* (%)]	295 (26)	42 (32)	279 (37)	163 (34)	<0.001
Dyslipidemia	[*n* (%)]	104 (9)	11 (8)	90 (12)	37 (8)	0.059

Low SD						
*N*		4085	339	2041	1101	
Mean age	(years)	65.4 ± 7.3	64.1 ± 7.5^b^	66.0 ± 7.1^b^	64.9 ± 7.7^d^	<0.001
Female	[*n* (%)]	2263 (55)	138 (41)	944 (46)	513 (47)	<0.001
BMI	(kg/m2)	22.6 ± 2.9	23.3 ± 3.0^a^	23.7 ± 3.1^a^	24.3 ± 3.3^a^	<0.001
Current smoker	[*n* (%)]	626 (15)	58 (17)	352 (17)	209 (19)	0.020
Habitual drinker	[*n* (%)]	1684 (41)	174 (51)	949 (47)	493 (45)	<0.001
Hypertension	[*n* (%)]	1401 (34)	164 (48)	982 (48)	604 (55)	<0.001
Antihypertensive medication	[*n* (%)]	596 (15)	58 (20)	430 (21)	230 (21)	<0.001
Dyslipidemia	[*n* (%)]	435 (11)	30 (9)	253 (12)	112 (10)	0.074

Samples in the highest quartile were considered as having high SBP SDs and those in the remaining three quartiles as having low SBP SDs; BMI, body mass index; DM, diabetes; NGT, normal glucose tolerance; IFG, impaired fasting glucose; IGT, impaired glucose tolerance; SD, standard deviation; ^a^*P* < 0.001 versus NGT; ^b^*P* < 0.005 versus NGT; ^c^*P* < 0.01 versus NGT; ^d^*P* < 0.05 versus NGT.

**Table 2:** Blood pressure measurements of participants with normal glucose tolerance, impaired glucose metabolism, and diabetes.

		NGT	IFG	IGT	DM	*P*
*N*		5235	472	2795	1586	
Number of visits		5.5 ± 0.7	5.5 ± 0.8	5.5 ± 0.7	5.4 ± 0.8^a^	<0.001
Mean SBP	(mmHg)	129.7 ± 12.9	134.4 ± 13.0^a^	134.3 ± 12.8^a^	136.1 ± 13.3^a^	<0.001
Mean DBP	(mmHg)	75.5 ± 7.3	77.7 ± 7.2^a^	77.5 ± 7.2^a^	78.1 ± 7.4^a^	<0.001
SD SBP	(mmHg)	9.5 ± 3.8	10.1 ± 3.9^b^	10.1 ± 4.1^a^	10.3 ± 4.2^a^	<0.001
SD DBP	(mmHg)	6.3 ± 2.4	6.6 ± 2.5^b^	6.5 ± 2.5^a^	6.6 ± 2.6^a^	<0.001
CV SBP	(%)	7.4 ± 2.8	7.5 ± 2.8	7.5 ± 2.9^c^	7.6 ± 3.0^c^	0.032
CV DBP	(%)	8.4 ± 3.1	8.6 ± 3.0	8.4 ± 3.1	8.5 ± 3.3	0.565

Classified by SBP SD						

High SD						
*N*		1150	133	754	485	
Number of visits		5.5 ± 0.7	5.4 ± 0.8	5.5 ± 0.7	5.5 ± 0.7	0.105
Mean SBP	(mmHg)	134.6 ± 13.3	140.0 ± 12.8^a^	138.3 ± 12.6^a^	139.8 ± 13.3^a^	<0.001
SD SBP	(mmHg)	15.0 ± 2.7	14.8 ± 2.4	15.3 ± 3.0	15.3 ± 2.7	0.058
Low SD						
*N*		4085	339	2041	1101	
Number of visits		5.5 ± 0.7	5.5 ± 0.8	5.5 ± 0.7	5.4 ± 0.8^a^	<0.001
Mean SBP	(mmHg)	128.3 ± 12.5	132.2 ± 12.5^a^	132.8 ± 12.6^a^	134.5 ± 13.0^a^	<0.001
SD SBP	(mmHg)	8.0 ± 2.4	8.2 ± 2.5	8.2 ± 2.4^b^	8.1 ± 2.4	0.047

Samples in the highest quartile were considered as having high SBP SDs and those in the remaining three quartiles as having low SBP SDs. CV, coefficients of variation; DM, diabetes; DBP, diastolic blood pressure; NGT, normal glucose tolerance; IFG, impaired fasting glucose; IGT, impaired glucose tolerance; SBP, systolic blood pressure; SD, standard deviation; ^a^*P* < 0.001 versus NGT; ^b^*P* < 0.01 versus NGT; ^c^*P* < 0.05 versus NGT.

**Table 3:** Univariate and multivariate OR for high BP variability.

	Univariate			Multivariate		
High SD of SBP	OR	95% CI	*P*	OR	95% CI	*P*

NGT	1			1		
IFG	1.39	(1.13–1.72)	0.003	1.39	(1.12–1.73)	0.003
IGT	1.31	(1.18–1.46)	<0.001	1.26	(1.13–1.41)	<0.001
DM	1.56	(1.38–1.77)	<0.001	1.54	(1.35–1.76)	<0.001

High SD of DBP	OR	95% CI	*P*	OR	95% CI	*P*

NGT	1			1		
IFG	1.13	(0.91–1.40)	0.275	1.05	(0.84–1.30)	0.655
IGT	1.12	(1.00–1.24)	0.045	1.04	(0.93–1.16)	0.477
DM	1.20	(1.05–1.36)	0.006	1.11	(0.97–1.26)	0.132

High CV of SBP	OR	95% CI	*P*	OR	95% CI	*P*

NGT	1			1		
IFG	1.09	(0.87–1.35)	0.436	1.14	(0.92–1.42)	0.226
IGT	1.12	(1.01–1.24)	0.043	1.15	(1.03–1.28)	0.015
DM	1.28	(1.13–1.45)	<0.001	1.35	(1.19–1.54)	<0.001

High CV of DBP	OR	95% CI	*P*	OR	95% CI	*P*

NGT	1			1		
IFG	1.06	(0.85–1.31)	0.588	1.07	(0.86–1.33)	0.516
IGT	1.02	(0.92–1.14)	0.694	1.02	(0.91–1.13)	0.762
DM	1.04	(0.91–1.18)	0.543	1.05	(0.92–1.20)	0.431

Multivariate model included age, gender, body mass index, smoking, drinking, taking antihypertensive medications, dyslipidemia, and number of visits; High SDs of SBP and DBP and high CVs of SBP and DBP were defined as the highest quartile; CV, coefficients of variation; DM, diabetes; DBP, diastolic blood pressure; NGT, normal glucose tolerance; IFG, impaired fasting glucose; IGT, impaired glucose tolerance; OR, odds ratio; SBP, systolic blood pressure; SD, standard deviation.
